# Conventional osteosarcoma of the mandible successfully treated with radical surgery and adjuvant chemotherapy after responding poorly to neoadjuvant chemotherapy: a case report

**DOI:** 10.1186/s13256-017-1386-0

**Published:** 2017-08-02

**Authors:** Yutaro Kimura, Kei Tomihara, Hidetake Tachinami, Shuichi Imaue, Kenji Nakamori, Kumiko Fujiwara, Kayo Suzuki, Taketoshi Yasuda, Shigeharu Miwa, Eiji Nakayama, Makoto Noguchi

**Affiliations:** 1 Department of Oral and Maxillofacial Surgery, Graduate School of Medicine and Pharmaceutical Sciences for Research, University of Toyama, 2630 Sugitani, Toyama city, Toyama 930-0194 Japan; 2 Department of Oral and Maxillofacial Surgery, Naha City Hospital, 2-31-1 Furujima, Naha, Okinawa 902-8511 Japan; 3 Department of Orthopaedic Surgery, Graduate School of Medicine and Pharmaceutical Sciences for Research, University of Toyama, 2630 Sugitani, Toyama city, Toyama 930-0194 Japan; 4 Department of Diagnostic Pathology, Graduate School of Medicine and Pharmaceutical Sciences for Research, University of Toyama, 2630 Sugitani, Toyama city, Toyama 930-0194 Japan; 5 Division of Oral and Maxillofacial Radiology, Department of Human Biology and Pathophysiology, School of Dentistry, Health Sciences University of Hokkaido, 1757 Kanazawa, Tobetsu, Ishikari, 061-0293 Japan

**Keywords:** Conventional osteosarcoma, Mandible, Head and neck, Chemotherapy

## Abstract

**Background:**

Osteosarcoma, the most common primary bone malignancy, has an extremely poor prognosis and a high rate of local recurrence and distal metastases. Because osteosarcomas of the head and neck region are rare, accounting for less than 10% of all osteosarcoma cases, limited information is available about their treatment and prognosis. Because of the high rate of distal metastases associated with extragnathic osteosarcoma, surgery combined with chemotherapy is currently considered essential in its treatment. However, the role of chemotherapy has not been well elucidated in the treatment of head and neck osteosarcoma because of the rarity of this condition.

**Case presentation:**

In this report, we present the case of a 58-year-old Japanese woman with osteosarcoma of the mandible that was treated with radical surgery combined with neoadjuvant and adjuvant chemotherapy. Because the tumor showed rapid growth during neoadjuvant chemotherapy, neoadjuvant chemotherapy was suspended and surgical resection was performed, followed by adjuvant chemotherapy. No evidence of local recurrence and distal metastasis was found 14 months after initial treatment. Local control is considered a principal prognostic factor for head and neck osteosarcoma.

**Conclusions:**

Wide surgical excision should be considered a primary goal even during neoadjuvant chemotherapy, especially in cases that respond poorly to neoadjuvant chemotherapy.

## Background

Osteosarcoma is a high-grade primary bone malignancy with a high rate of metastasis and local recurrence [1, 2]. The most common anatomical sites affected are the long bones of the limbs, particularly in the knee region [1, 2]. In contrast, osteosarcomas of the head and neck region are rare, accounting for less than 10% of all osteosarcomas [3–5]. Although osteosarcomas of long bones commonly occur in the second decade of life during bone growth [1, 2], osteosarcomas of the head and neck generally occur later, peaking in the second, third, and fourth decades of life [3–5]. Based on the 2013 World Health Organization (WHO) classification of bone tumors [6], osteosarcomas are histologically classified into different histologic subtypes, including conventional, telangiectatic, small-cell, low-grade central, secondary, periosteal, parosteal, and high-grade surface. Moreover, conventional osteosarcoma, the most frequent type of osteosarcoma, is classified into three subtypes: osteoblastic, chondroblastic, and fibroblastic osteosarcoma [1]. The occurrence rate of distant metastases is approximately 80%, and the 5-year survival rate is 20 to 30% after surgery alone without neoadjuvant and adjuvant chemotherapy. Moreover, 20% of patients with osteosarcoma have distant metastases when the primary tumor is newly diagnosed [7]. Therefore, neoadjuvant and/or adjuvant chemotherapy is currently deemed an essential adjunct to surgical treatment, especially in the management of high-grade osteosarcoma [7, 8]. In fact, neoadjuvant and adjuvant chemotherapy in combination with surgery dramatically improves clinical outcomes in extragnathic osteosarcoma, such as an increase in the disease-free survival rate from 10 to 20% to >60% [7, 8]. Although the vast majority of head and neck osteosarcomas are high-grade conventional osteosarcomas [9–12], they are highly heterogeneous compared with extragnathic osteosarcoma. Low-grade osteosarcomas, such as parosteal and central osteosarcomas, and intermediate-grade osteosarcomas, such as periosteal osteosarcomas, sometimes overlap morphologically and clinically with benign bone diseases. This overlap occurs more frequently with head and neck osteosarcomas than with extragnathic osteosarcomas [13]. Moreover, head and neck osteosarcomas are less aggressive and have a better prognosis with surgery alone than extragnathic osteosarcoma does, in particular in low-grade osteosarcomas such as parosteal and central, which occur relatively more frequently than extragnathic osteosarcoma does [13–18]. Therefore, the management of head and neck osteosarcoma is not standardized and varies widely among institutions.

In the present case report, we describe a case of high-grade conventional osteosarcoma of the mandible that was successfully treated with a combination of surgery, and neoadjuvant and adjuvant chemotherapy.

## Case presentation

**Fig. 1:**
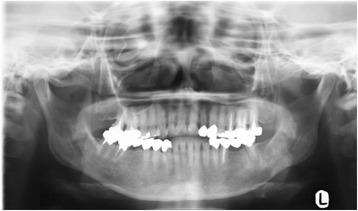
A panoramic radiograph showing a loss of the lamina dura and a well-defined periapical radiolucent lesion around the root apex of tooth #37, and an irregular bordered radiolucent lesion involving the left body of the mandible extending from the tooth #35 to #38 region, with an indistinct cortical margin and ill-defined cortical borders of the inferior alveolar nerve canal

**Fig. 2:**
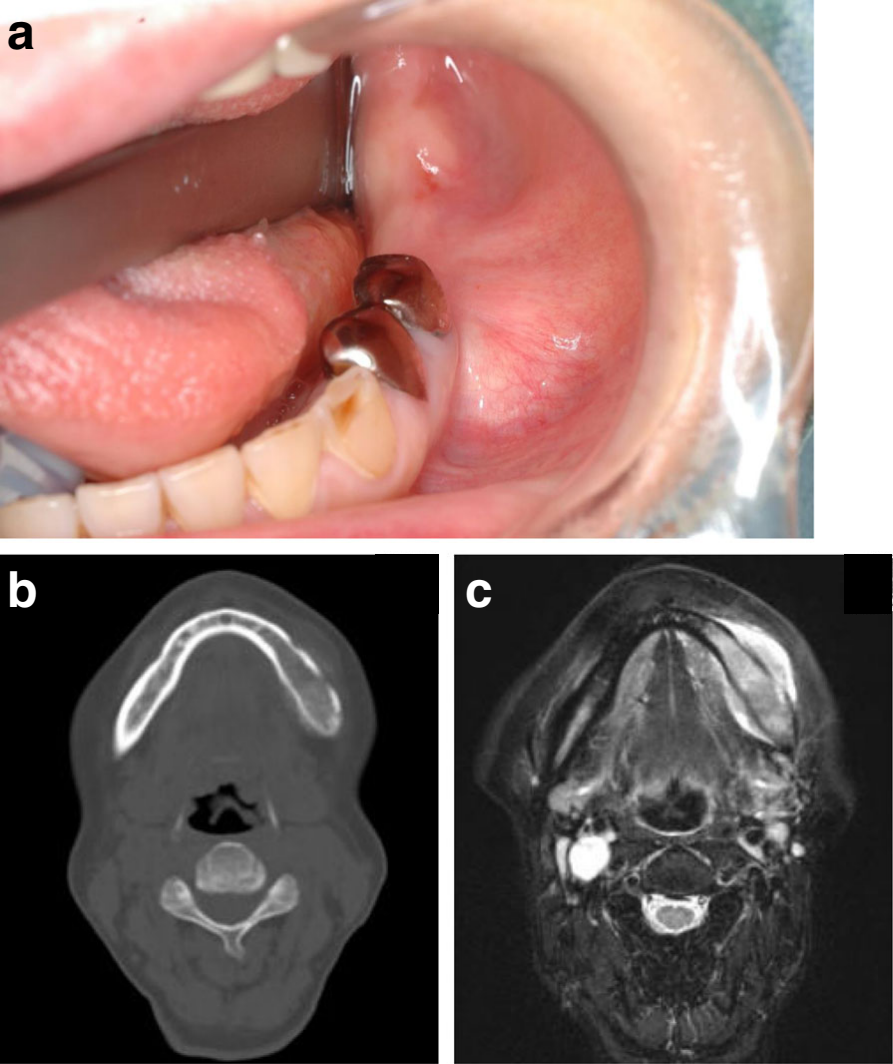
**a** An intra-oral photograph showing a slight diffuse swelling of the mandible with normal appearance of the overlying mucosa. **b** A computed tomography scan acquired before neoadjuvant chemotherapy showing the lesion on the left mandible with an indistinct cortical margin and small bony spicules. **c** A fat-saturated T2-weighted image from magnetic resonance imaging performed before neoadjuvant chemotherapy showing a high intensity lesion on the left mandible, with peritumoral soft tissue enhancement

**Fig. 3:**
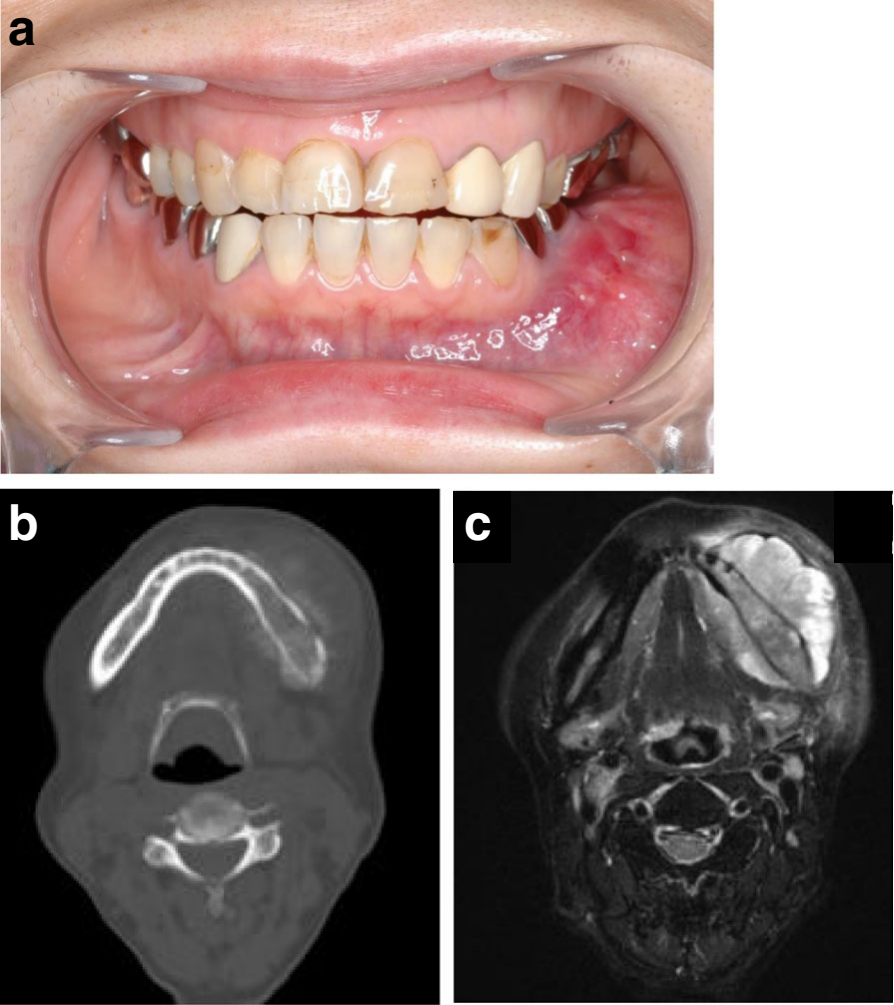
**a** An intra-oral photograph after neoadjuvant chemotherapy showing expansive diffuse swelling of the mandible with the erythematous appearance of the overlying mucosa. **b** A computed tomography scan acquired after neoadjuvant chemotherapy showing the lesion on the left mandible accompanied with the sunburst appearance of marked osteoid formations. **c** A fat-saturated T2-weighted image from magnetic resonance imaging performed after neoadjuvant chemotherapy showing a high intensity lesion on the left mandible, with prominent peritumoral soft tissue enhancement

**Fig. 4:**
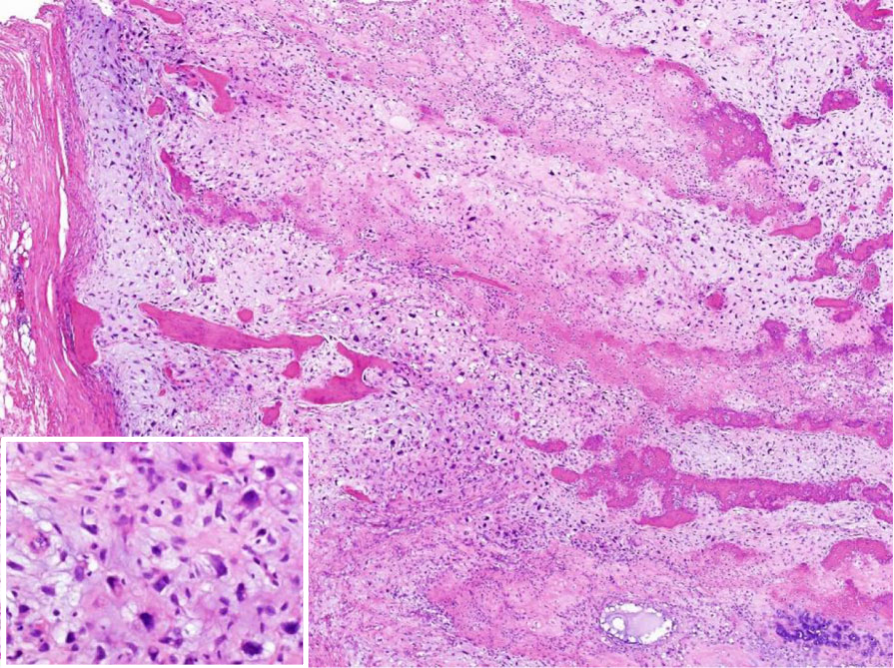
Microscopic histopathology of the hematoxylin and eosin-stained tumor specimen. Photomicrograph of the histological specimen showing conventional osteosarcoma composed of sarcomatous tumor cells that produced both osteoid and immature bone, and chondroid matrices. Insert showing a high power view of severely atypical cells of the lesion

A 58-year-old Japanese woman complaining of pain and numbness in her left mandible was referred to our hospital in 2014. For a couple of months prior to her visit, she had been aware of an abnormal sensation in her left mandible, which gradually progressed to mild pain and numbness. She visited a general dental practitioner, who diagnosed her condition as osteomyelitis and referred her to our department. Her medical and family histories were unremarkable. On initial assessment, no obvious systemic symptoms were evident. A panoramic radiograph showed a widening of the periodontal ligament space, periapical bone loss in tooth #37, and a diffuse radiolucent lesion involving the left body of her mandible, with an indistinct cortical margin and ill-defined cortical borders of the inferior alveolar nerve canal (Fig. 1). Moreover, the radiograph also showed that tooth #37 had previously been treated endodontically. Therefore, a diagnosis of apical periodontitis was suggested and endodontic treatment was performed; however, her symptoms were not relieved. Consequently, a neoplastic lesion was highly suspected and findings of a biopsy of the apical tissue after extraction of tooth #37 resulted in a histopathological diagnosis of tissue inflammation. However, after the biopsy, a gradual progressive swelling of the left mandible occurred (Fig. 2a). Computed tomography (CT) showed an enhanced lesion on the left mandible, and magnetic resonance image (MRI) showed abnormally high-intensity signal in the bone marrow, with surrounding soft tissue mass (Fig. 2b, c). Therefore, we performed an incisional biopsy of the swollen area, the findings of which resulted in a histopathological diagnosis of osteoblastic-type osteosarcoma of the mandible. She was then scheduled for radical surgery combined with neoadjuvant and adjuvant chemotherapy based on the regimen used in a multi-institutional clinical study of neoadjuvant chemotherapy in extragnathic osteosarcoma (NECO study) in Japan [19]. In the NECO study, neoadjuvant chemotherapy consisted of two courses of high-dose (HD) methotrexate (MTX) followed by a course of cisplatin (CDDP) and adriamycin (ADR) as phase I chemotherapy. After phase I chemotherapy was completed, the response to induction chemotherapy was evaluated. If the treatment response was assessed as complete response (CR), partial response (PR), or stable disease (SD), four courses of HD-MTX and a course of CDDP and ADR were administered. In contrast, if the treatment was assessed on the basis of the response as “not effective, with progressive disease (PD),” the chemotherapy regimen was changed to HD ifosfamide (IFO). Toxic effects during chemotherapy were graded according to the Common Terminology Criteria for Adverse Events Version 4.0. Following neoadjuvant chemotherapy, tumors were assessed using response evaluation criteria in solid tumors (RECIST) after determining their sizes using CT and MRI. In the current patient, the swelling increased rapidly during the phase I neoadjuvant chemotherapy (Fig. 3a). CT and MRI also revealed marked progression of the lesion (Fig. 3b, c), and laboratory data showed marked elevation of serum alkaline phosphatase. On the basis of these data, we assessed the response to neoadjuvant chemotherapy as not effective, with PD. Therefore, the neoadjuvant chemotherapy was suspended and radical surgery took precedence before the lesion grew to an unresectable size. She was then treated with radical surgery consisting of a hemimandibulectomy and reconstruction using a free vascularized latissimus dorsi pedicle flap and rigid titanium reconstruction plate. On histologic examination, the tumor was composed of stellate cells, which were large and atypical (Fig. 4). Highly atypical cells produced osteoid and immature bone. Moreover, chondroid matrices were also observed. Taken together, these findings indicated that the therapeutic response was poor, assessed as grade 0 (tumor necrosis area <90%). On postoperative day 25, adjuvant chemotherapy was started. Adjuvant chemotherapy was also performed in accordance with the NECO study regimen, with slight modifications. The adjuvant chemotherapy regimen included two courses of HD-IFO followed by a course of CDDP and ADR, and the same regimen was repeated for a total of three cycles. During chemotherapy, hematologic toxicities, grade 4 leukopenia, and thrombocytopenia were detected and the frequency of febrile neutropenia increased, requiring red blood cell and platelet transfusions and the use of granulocyte-colony stimulating factor. The treatment schedule and our patient’s clinical course are summarized in the Table 1. No evidence of local recurrence and distant metastasis was found at 14 months follow-up after initial treatment.

**Table 1:** Clinical course and treatment schedule

	HD-MTX	HD-MTX	CDDP+ADR	Surgery	IFO	IFO	CDDP+ADR	IFO	IFO	CDDP+ADR	IFO	IFO
Leukopenia	-	-	G1		G4	G4	G3	G4	G4	G4	G4	G4
Neutropenia	-	G2	G2		G4	G4	G2	G4	G4	G4	G4	G4
Platelet	-	-	-		-	-	-	-	-	-	G3	G4
Vomiting	-	-	-		G2	G2	-	G1	G1	-	G2	G2
Anemia	-	-	-		-	-	-	-	-	-		G4

*ADR* adriamycin, *CDDP* cisplatin, *G1* Grade 1, *G2* Grade 2, *G3* Grade 3, *G4* Grade 4, *HD* high-dose, *IFO* ifosfamide, *MTX* methotrexate. Regimen-related toxicity was graded according to the common terminology criteria for adverse events (CTCAE)

## Discussion

Although no consensus exists regarding the efficacy of chemotherapy in head and neck osteosarcoma, a few studies have shown that chemotherapy in neoadjuvant and adjuvant setting improves survival in patients with head and neck osteosarcoma [16, 20]. However, a more recent study failed to show a benefit of chemotherapy in head and neck osteosarcoma [10]. The principal prognostic factor in head and neck osteosarcoma is complete tumor resection with negative margins in radical surgery [10, 16, 20–23]. A distinct feature of surgery in head and neck lesions is the complexity of head and neck anatomy, which increases the potential for functional and cosmetic impairment due to ablation surgery compared with the potential at other tumor sites.

Although the width of surgical margins remains controversial, and clear bony margins of 2 cm have been reported after effective preoperative chemotherapy [24], the safe recommended margin in osteosarcoma of the extremities is 3 cm [13]. In head and neck osteosarcomas, a clear surgical margin of at least 1 cm has been recommended because of the anatomical limitations that prevent wide resection [25]. However, this margin may still be insufficient, and probably increases the risk of local recurrence. Therefore, 2-cm bony margins and at least 5-mm soft tissue margins have been recommended, assuming that reconstructive surgery is possible [13].

In the present case, because the tumor was unresponsive and showed rapid growth during neoadjuvant chemotherapy, this therapy was suspended and radical surgery took precedence. Local control is currently the most important prognostic factor for survival in head and neck osteosarcoma. Therefore, to avoid missing the optimal time window for performing complete surgical excision with clear margins, clinicians should meticulously assess the response to neoadjuvant chemotherapy and not hesitate to suspend this treatment if the response is inadequate.

The most critical issue in the future development of novel treatment strategies for osteosarcoma in the head and neck is the circumvention of therapy-resistant osteosarcoma. To accomplish this, biomarkers predicting the response to chemotherapy urgently need to be identified, especially for the treatment of patients who respond poorly to chemotherapy. Nevertheless, limited data are available about the mechanism by which chemoresistance develops in head and neck osteosarcoma. Various processes have been implicated in the mechanism underlying the development of chemoresistance in extragnathic osteosarcoma, including altered cell cycle [26], reduced intracellular drug accumulation [27], drug inactivation [28], increased drug detoxification [29], altered deoxyribonucleic acid (DNA) repair mechanism [30], altered apoptosis signaling [31], autophagy [32], micro ribonucleic acid (RNA) dysregulation [33], and cancer stem cells [34]. A thorough understanding of these processes is important for predicting treatment response and for developing novel treatment strategies to prevent the emergence of therapy-resistance in head and neck osteosarcoma. Further studies will be necessary to elucidate the clinical impact of chemotherapy in the management of head and neck osteosarcoma, and to clarify the role of chemoresistance in disease relapse, progression, and poor prognosis for survival.

## Conclusions

Because of the high rate of distal metastases, surgery combined with chemotherapy is currently recommended as an essential treatment for extragnathic osteosarcoma. Local control has been suggested to be the principal prognostic factor for head and neck osteosarcoma; therefore, the achievement of wide surgical excision should be the primary goal, even during neoadjuvant chemotherapy, especially in patients who respond poorly to neoadjuvant chemotherapy.

Further studies are necessary to clarify the clinical impact of chemotherapy on tumor control or better prognosis for survival which will contribute to the establishment of the role of chemotherapy in the management of head and neck osteosarcoma.

## Abbreviations

ADR: Adriamycin
CDDP: Cisplatin
CR: Complete response
CT: Computed tomography
DNA: Deoxyribonucleic acid
HD: High-dose
IFO: Ifosfamide
MRI: Magnetic resonance image
MTX: Methotrexate
NECO: Neoadjuvant chemotherapy in extragnathic osteosarcoma
PD: Progressive disease
PR: Partial response
RECIST: Response evaluation criteria in solid tumors
RNA: Ribonucleic acid
SD: Stable disease
WHO: World Health Organization
